# Molecular and developmental consequences of heat stress on the bovine oocyte and embryo competence

**DOI:** 10.1590/1984-3143-AR2026-0067

**Published:** 2026-07-27

**Authors:** Ghyslaine Giselle Ramírez, Nico Graham Menjivar, Samuel Gebremedhn, Ahmed Gad, Eva Held-Hoelker, Michael Hoelker, Genet Zewdie, Dawit Tesfaye

**Affiliations:** 1 Animal Reproduction and Biotechnology Laboratory – ARBL, Department of Biomedical Sciences, Colorado State University, Fort Collins, CO, United States of America; 2 Stanford Fertility and Reproductive Health Services, Stanford Medicine Children’s Health, Sunnyvale, CA, United States of America; 3 J.R. Simplot Company, Boise, ID, United States of America; 4 Animal Reproduction and Biotechnology Laboratory – ARBL, Department of Clinical Sciences, Colorado State University, Fort Collins, CO, USA; 5 Department of Animal Breeding, Institute of Animal Sciences, University of Bonn, Bonn, Germany; 6 Department of Animal Science, Biotechnology and Reproduction of Farm Animals, Georg-August-University Goettingen, Göttingen, Germany; 7 Addis Abeba University, Addis Abeba, Ethiopia

**Keywords:** heat stress, reproduction, ovary, oocyte and embryo

## Abstract

Seasonal heat stress (HS) is a pervasive environmental challenge with profound consequences for female reproductive physiology, affecting ovarian function, oocyte maturation, and early embryonic development. At the ovarian level, HS disrupts follicular growth, impairs steroidogenesis, and compromises granulosa cell function, thereby creating a suboptimal microenvironment that reduces oocyte competence. In oocytes, HS induces oxidative stress, mitochondrial dysfunction, endoplasmic reticulum (ER) stress, spindle abnormalities, chromosomal missegregation, and persistent epigenetic alterations. These disruptions extend into early embryonic development, where redox imbalance, apoptosis, ER stress, and altered lineage allocation reduce cleavage and blastocyst formation, compromise trophectoderm and inner cell mass integrity, and impair implantation potential. Maternal heat exposure further exacerbates embryonic vulnerability by altering the oviductal and uterine environment, reducing embryotrophic factors and antioxidant defenses, and ultimately influencing offspring phenotype and fertility, potentially across generations. Accordingly, this review aims to synthesize current knowledge on the physiological and molecular impacts of heat stress on ovarian function, oocyte maturation, and early embryonic development. To this end, we consider studies conducted under both in vivo and in vitro conditions, highlighting shared and distinct mechanisms of thermal stress at the organ and cellular levels to identify potential targets for intervention.

## Introduction

Global warming induced seasonal thermal stress is continuing to rise (Hansen et al., 2006) with an exponential increase in the number of heat days in different regions of the world (Davariashtiyani et al., 2023). Extreme HS during summer disrupts various reproductive processes ranging from ovarian functionality to pronounced depression of conception rate in beef and dairy cows worldwide (Davariashtiyani et al., 2023). HS exerts a significant impact on animal productivity, health, reproduction, and overall welfare. The situation is aggravated for dairy cows, as the sector is under tremendous pressure to increase milk production amid rising demand worldwide for milk and milk products. The environmental HS and endogenous heat production due to high metabolic rate in dairy cow’s compromise animal reproductive performance, which is an essential component for farm productivity (Tao et al., 2020).

The impact of seasonal thermal stress on mammalian female reproduction is multifaceted. One of the most affected dynamic organs is the ovary, and its follicular microenvironment. Ovaries are responsible for safeguarding female gametes and secreting ovarian steroids that ultimately regulate reproductive functions. HS disrupts reproductive function through physiological, behavioral, and endocrine alterations that directly affect the hypothalamic–pituitary–ovarian (HPO) axis. Ultrasonographic studies have demonstrated that HS alters ovarian follicular dynamics and impairs follicular growth rate and prolongs follicular wave duration, indicating compromised follicular function (Dovolou et al., 2023). HS increased the number of midsize follicles thereby reducing follicular dominance, which is accompanied by decreased estradiol and inhibin secretions and increased circulating FSH levels (Parker et al., 2003; Wolfenson et al., 1995a). As the functional unit of the ovary, the impact of HS on the follicle has a far-reaching effect on the oocyte and the functionality of the surrounding somatic cells (granulosa and theca cells).

In addition to seasonal measurements on the impact of thermal stress on oocyte maturation, several in vitro experiments using temperatures up to 43°C to induce HS in oocytes indicated a significant impact on oocyte growth (Kawano et al., 2022), reduced mitochondrial membrane potential (MMP), increased accumulation of reactive oxygen species (ROS), oxidative stress, and induced apoptosis (Feng et al., 2024; Lee et al., 2023; Roth and Hansen, 2005; Wrzecińska et al., 2023). Taken together, the detrimental effects of HS are observed across in vivo and in vitro models, which significantly impair oocyte quality by disrupting both nuclear and cytoplasmic maturation, increasing chromosomal and spindle abnormalities, elevating oxidative stress and apoptosis, and compromising subsequent embryo development.

Several studies have demonstrated the carryover effects of HS exposure during in vitro oocyte maturation on the developmental competence and molecular architecture of the resulting blastocysts. Specifically, altered MMP, reduced ATP levels, and decreased mtDNA copy number have been reported in blastocysts derived from HS-exposed oocytes (Feng et al., 2024; Miętkiewska et al., 2022). HS is closely associated with reduced reproductive performance in cattle, in part due to its detrimental effects on blastocyst formation, implantation rates, and increased embryonic mortality. The severity of these impairments is largely stage-specific, with early developmental stages, particularly cleavage and blastocyst formation, being most susceptible. In this literature review, we systematically summarize findings from both in vitro and in vivo studies that evaluate the molecular, functional, and developmental impacts of environmental thermal stress on ovarian and follicular function, oocyte maturation, and early embryonic development.

## The impact of thermal stress on ovarian function

### Impact of heat stress on follicular growth and dominance

Summer HS adversely affects animal performance and health, disrupting follicular growth (Wolfenson et al., 1997), corpus luteum (CL) function, preimplantation embryo development, and the uterine environment and receptivity. A particularly pronounced effect of HS occurs during follicular development. HS impairs the growth and function of small antral follicles, thereby disrupting the formation of the preovulatory dominant follicle and compromising both ovulation and CL formation (Roth et al., 2000; Wolfenson et al., 1995b). The vulnerability of ovarian follicles to heat stress depends on the developmental stage. Preantral, primary, and secondary follicles are susceptible to elevated temperatures under in vitro conditions, with secondary follicles exhibiting the highest sensitivity, followed by early secondary follicles (Aguiar et al., 2020). Notably, dairy cows exposed to elevated temperatures exhibited reduced diameters of dominant follicles during the first and second follicular waves, resulting in disrupted follicular dominance (Wolfenson et al., 1995a; Badinga et al., 1993; Wilson et al., 1998). This disruption is characterized by an increased number of mid-sized dominant follicles and the absence of a single dominant follicle, leading to a significant reduction in plasma inhibin concentration and an increase in follicle-stimulating hormone (FSH) levels (Roth et al., 2000). These alterations may explain the higher incidence of abnormal ovulation patterns and twinning observed during summer (Ryan and Boland, 1991). The impact of HS on follicular development is long-lasting; cows exposed to HS require at least four to six follicular waves to recover and produce developmentally competent oocytes after HS exposure (Hulshof et al., 1994; Roth et al., 2001).

### Impact of elevated temperature on follicular microenvironment

**Figure 1:**
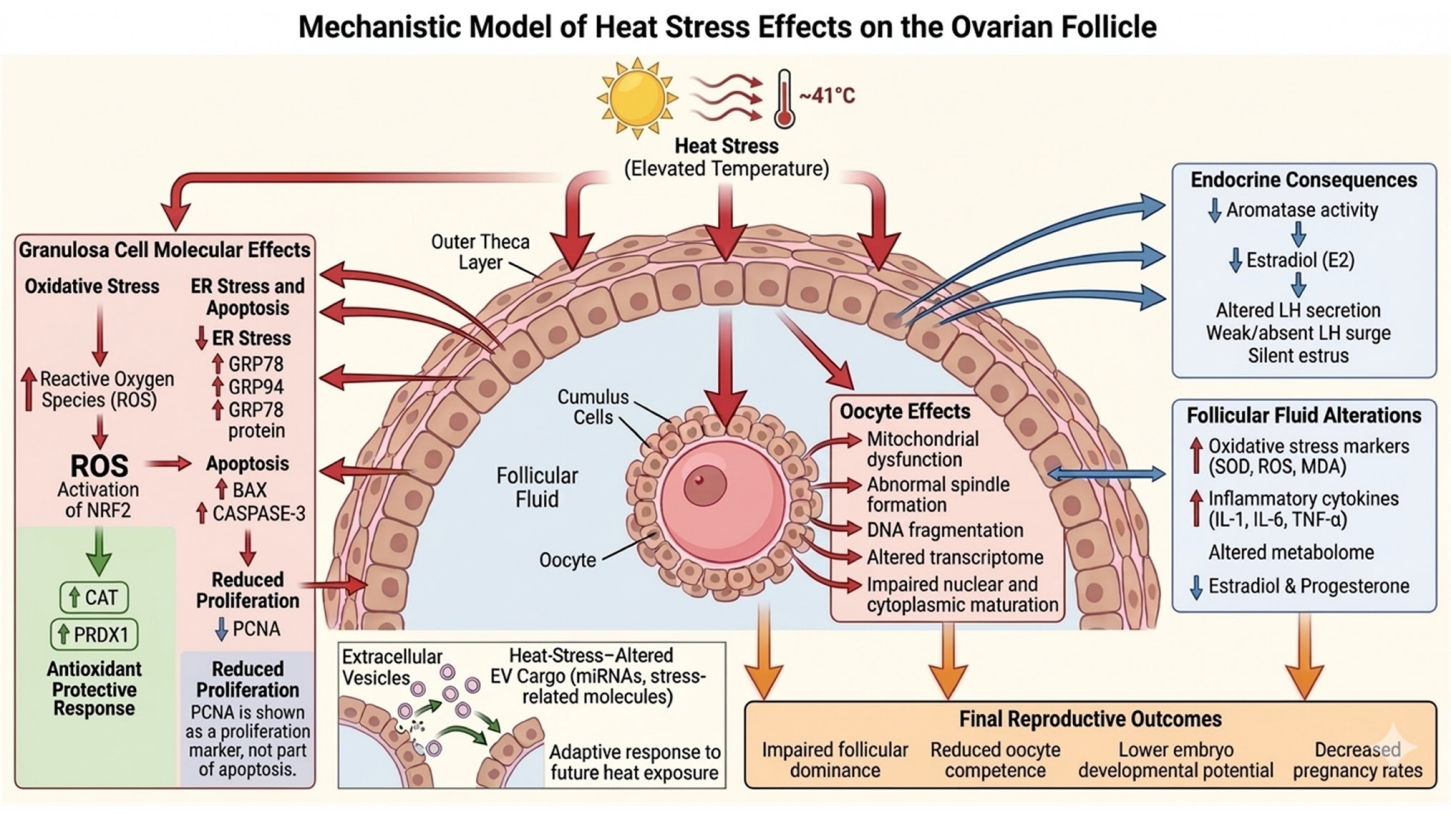
A model illustrating the molecular consequences of environmental heat stress (HS) on ovarian function and its subsequent impact on reproductive outcomes. Reduced fertility reflects the cumulative effects of HS on follicular cells (granulosa and theca cells), including disrupted steroidogenesis, increased oxidative stress, mitochondrial dysfunction, and altered gene expression, which collectively compromise follicular homeostasis. In parallel, HS perturbs extracellular vesicle (EV)-mediated molecular signaling, impairing the transfer of regulatory molecules (e.g., miRNAs, proteins, and lipids) and disrupting intercellular communication within the follicular microenvironment. These alterations, together with impaired oocyte growth, meiotic competence, and cytoplasmic maturation, ultimately result in reduced developmental competence, compromised embryo quality, impaired implantation potential, and decreased pregnancy rates.

A study performed in dairy cows to determine the impact of HS on the metabolic characteristics of the follicular fluid revealed that HS significantly changes the abundance of metabolites that are associated with oxidative stress, including SOD, GSH-Px, CAT, MDA, T-AOC, ROS, inflammatory factors including IL-1, IL-6, TNF-α, heat shock proteins, HSP70, and HSP90, and steroid hormones estradiol and progesterone (Li et al., 2025). The study further reported differential 1,544 metabolites in the follicular fluid of the heat-stressed group, which were mainly enriched in pathways such as steroid hormone biosynthesis, neuroactive ligand-receptor interactions, D-amino acid metabolism, tyrosine metabolism, phenylalanine metabolism, and tryptophan metabolism. Molecular analysis revealed that mural granulosa cells from heat-stressed cows had lower expression of key steroidogenic proteins, including StAR, LHR, aromatase (CYP19A1), and lower follicular estradiol levels, along with increased oxidative stress markers, including SOD1 and SOD2, and the apoptosis effector caspase‑3, which were positively correlated with oxidative stress-induced apoptosis (Kim et al., 2026). These results demonstrated that thermal stress induces coordinated disruptions in molecular, hormonal, and oxidative pathways within the ovarian microenvironment, ultimately impairing oocyte maturation and reducing fertility. The molecular consequences of thermal stress on ovarian follicular development, endocrine function, cellular proliferation, EV cargo composition, and subsequent impact on reproductive outcomes are summarized in Figure 1.

### Impact of heat stress on the function of follicular cells

**Figure 2:**
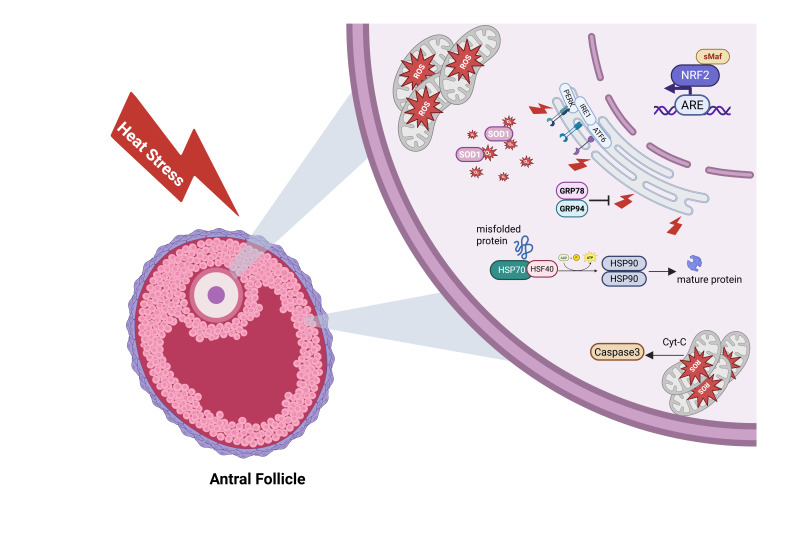
Molecular mechanisms underlying the response of antral follicle–enclosed somatic cells and oocytes to thermal stress. Heat stress induces oxidative stress, endoplasmic reticulum (ER) stress, and mitochondrial dysfunction in both somatic (granulosa and theca) cells and the oocyte. The primary line of defense, and among the most extensively studied pathways, includes the NRF2-mediated antioxidant response and the unfolded protein response (UPR), which act coordinately to restore cellular homeostasis by regulating redox balance, protein folding, and stress adaptation. When these protective mechanisms are insufficient or overwhelmed, persistent stress leads to apoptosis, impaired cellular communication, and compromised oocyte competence, ultimately affecting follicular integrity and developmental potential.

Granulosa cells are considered the most important follicular somatic cells that support the growth and development of oocytes, provide a suitable microenvironment that ensures the maturation and ovulation of developmentally competent oocytes, and secrete hormones (Edson et al., 2009). Recently, a field experiment on the transcriptomic profile of GCs derived from beef cows obtained during summer and winter seasons revealed distinct transcriptomic profiles and genes involved in pathways including cell adhesion molecules, chemokine signaling, cytokine-cytokine receptor interaction were enriched by genes down regulated in GCs derived from summer season, while genes upregulated are involved in protein processing and metabolic pathways (Joyce et al., 2024). This signifies the seasonal alteration in the gene expression profile of GCs in beef cows, which can potentially reflect seasonal variability in pregnancy success. Under in vitro conditions, several reports have shown adverse effects of HS on granulosa cell survival and the expression of key genes. Bovine GCs subjected to high temperatures (41°C) showed increased ROS at 24h, activating NRF2 and its antioxidant targets CAT and PRDX1. It also induced strong endoplasmic reticulum stress, marked by elevated GRP78 and GRP94 expression and increased GRP78 protein levels, along with upregulation of apoptotic genes BAX and CASPASE-3 (Alemu et al., 2018) (Figure 2).

Concurrently, HS reduced cell proliferation, reflected by decreased PCNA expression. Overall, elevated temperature triggered oxidative stress, ER stress, apoptosis, and reduced proliferation, prompting NRF2-mediated protective responses in bovine GCs (Alemu et al., 2018). Similarly, GCs subjected to in-vitro HS showed increased ROS, oxidized proteins, apoptosis, and elevated expression of heat shock proteins and antioxidants, alongside reduced cell viability (Gebremedhn et al., 2020). Interestingly, in the same study, exposure to higher temperature altered the expression of cellular miRNAs in GCs and the extracellular vesicles-mediated release of miRNAs in response to HS. The study further revealed that supplementing GCs cultures with EVs from heat-stressed cells promoted an adaptive response during subsequent heat exposure.

## Molecular impact of heat stress on oocyte maturation

HS during oocyte maturation reduces bovine oocyte developmental competence, cleavage rates, and blastocyst formation (Barros and Paula-Lopes, 2018; Feng et al., 2024). It triggers oxidative damage, ER dysfunction, and altered gene expression in oocytes as well as surrounding cumulus cells (Miętkiewska et al., 2022; Naranjo-Gómez et al., 2021). These changes have far-reaching consequences affecting the pre- and post-implantation phases of embryonic development (Feng et al., 2024;). Thermal stress adversely affects both nuclear and cytoplasmic maturation processes, frequently leading to aberrant spindle architecture, impaired mitochondrial function, promoting DNA fragmentation, and contributing to diminished embryo developmental potential (Aroyo et al., 2007).

### Redox imbalance in oocytes under heat stress

At the cellular level, HS is commonly modeled at 40–41.5°C during in vitro maturation (IVM) and has been shown to disrupt mitochondrial distribution and alter the expression of mitochondrial genes involved in the respiratory chain in metaphase II (MII) oocytes (Gendelman and Roth, 2012). Furthermore, elevated temperatures increase mitochondrial workload and electron transport chain leakage, elevating mitochondrial ROS in germinal vesicle and MII oocytes, overwhelming antioxidant defenses like SOD and GSH-Px (Feng et al., 2024; Lang et al., 2024). In bovine IVM at 41°C, mitochondrial ROS levels in oocytes reach those observed following direct oxidant exposure, while glutathione buffering capacity becomes compromised (Held-Hoelker et al., 2025; Payton et al., 2018). Several studies have shown that ROS surges during IVM at 41°C, with NRF2 pathway activation as a compensatory response, though insufficient to prevent apoptosis. Moreover, this leads to lipid peroxidation, protein oxidation, and DNA damage, reducing mitochondrial membrane potential, ATP levels, and mtDNA copy number (Payton et al., 2018; Tariq et al., 2025). Redox imbalance has been shown to go along with an increased expression of pro-apoptotic genes, while the expression of anti-apoptotic genes decreases (Held-Hoelker et al., 2025; Itami et al., 2018). If adaptive antioxidant responses like SOD and GSH-Px fail, this pushes oocytes towards apoptosis (Cao and Kaufman, 2014; Latham, 2015; Zhao et al., 2017). Consequently, the accumulation of excessive ROS and chronic ER stress caused by elevated temperatures leads to spindle abnormalities, errors in chromosome segregation, and defects in the cytoskeleton in oocytes, thereby reducing their developmental competence (Payton et al., 2018; Song et al., 2024).

### ER stress response in oocytes exposed to thermal stress

In bovine and other mammalian oocytes, elevated mitochondrial ROS disturbs protein folding in the ER and calcium homeostasis, triggering the UPR and, if unresolved, induce apoptosis (Cao and Kaufman, 2014; Held-Hoelker et al., 2025; Payton et al., 2018; Zhao et al., 2017). The ER and mitochondria physically interact via mitochondria‑associated membranes (MAMs), which regulate Ca^2+^ transfer and apoptotic signaling in oocytes (Lin et al., 2021; Zhao et al., 2017). In mouse oocytes, increased MAM abundance and higher IP3R1 and PACS‑2 expression enhance ER–mitochondria coupling, sensitizing mitochondria to ER Ca^2+^ release and promoting ROS‑linked dysfunction (Zhao et al., 2017). These contacts create microdomains in which mitochondrial ROS and Ca^2+^ flux can rapidly affect the ER redox state and vice versa (Cao and Kaufman, 2014; Lin et al., 2021). Furthermore, redox imbalance fuels ER stress through mitochondrial-ER contacts, amplifying UPR and epigenetic disruptions. In agreement with this, antioxidant interventions (e.g., HO-1) or chaperone supplementation (HSP70) have been shown to partially rescue maturation rates (Divvela et al., 2025; Wang et al., 2019). Disturbed UPR mechanisms cause misfolded protein buildup, which, in turn, triggers ER stress markers, such as GRP78/BiP sequestration, in bovine oocytes and cumulus cells, compromising Ca^2+^ homeostasis and maternal mRNA storage. Resulting effects include spindle abnormalities and impaired blastocyst formation (Alemu et al., 2018; Lin et al., 2019). Elevated temperatures increase mitochondrial ROS, further oxidizing ER luminal and membrane proteins, disrupt Ca^2+^ pumps and channels, and exacerbate ER Ca^2+^ depletion, ultimately leading to the accumulation of misfolded proteins (Cao and Kaufman, 2014; Latham, 2015; Zhao et al., 2017).

### Thermal stress-induced epigenetic alterations in oocytes

Beyond the immediate impact of elevated temperatures on the intrinsic quality of oocytes and embryos, HS induces epigenetic modifications, including alterations in chromatin remodeling and DNA methylation patterns, in both oocytes and early embryos (Barros and Paula-Lopes, 2018; Diaz et al., 2021; Zhu et al., 2021). HS has been shown to downregulate histone modifications (H1, H2A, H2B, and H4) and DNA methylation in both bovine oocytes and embryos (Diaz et al., 2021; Feng et al., 2024). Exposure to HS before fertilization induced the accumulation of H3K9me3 and HP1 in early embryos. Specifically, an increased accumulation of H3K9me3 was observed in the nuclei of four-cell and eight-cell stage embryos derived from heat-shocked oocytes (Diaz et al., 2025). Furthermore, a higher proportion of four-cell-stage embryos derived from heat-shocked oocytes showed increased HP1 fluorescence, suggesting abnormal chromatin compaction (Camargo et al., 2019). Recent reports indicate that both in vivo and in vitro HS altered DNA methylation patterns and reduced developmental competence in bovine embryos, evidencing stage-dependent differences and highlighting susceptibility at early cleavage stages (Diaz et al., 2025). Feng and colleagues showed that HS significantly reduced global DNA methylation (5mC) and hydroxymethylation (5hmC) levels at all stages from oocyte to blastocyst, with a minimum at the 8-cell stage and persistently lower 5hmC in thermal-stressed embryos (Feng et al., 2024). This might be explained by reduced mRNA levels of DNMT1, DNMT3A, DNMT3B, and histone H2A in blastocysts derived from heat-stressed oocytes, indicating an impaired active and maintenance methylation machinery. Consequently, HS during bovine oocyte maturation might leave a persistent molecular “imprint” that is clearly detectable in early embryos up to the blastocyst stage, particularly in terms of redox homeostasis, ER stress/UPR signaling, and epigenetic programming (Barros and Paula-Lopes, 2018; Feng et al., 2024; Kawano et al., 2025).

### Embryonic consequences of heat stress during maturation

It is important to understand that HS effects during maturation affect the vitality of the resulting embryos. Zygotes and cleavage-stage embryos derived from heat-stressed oocytes show increased ROS levels, along with impaired antioxidant capacity and reduced developmental competence (Divvela et al., 2025). In agreement with findings in oocytes, altered mitochondrial membrane potential, reduced ATP, and mtDNA copy number were also detected in the resulting blastocysts (Feng et al., 2024; Miętkiewska et al., 2022). Furthermore, antioxidant systems (SOD, CAT, GSH-Px, peroxiredoxins) are downregulated or functionally overwhelmed in heat-stressed ovaries, follicles, and embryos, resulting in increased apoptotic index and TUNEL-positive nuclei in morulae and blastocysts derived from heat-stressed oocytes or early embryos (Barros and Paula-Lopes, 2018; Feng et al., 2024; Naranjo-Gómez et al., 2021). Reduction of inner cell mass and compromised trophectoderm integrity, as well as altered expression of stress-related and developmentally important genes (e.g., HSPs, BAX/BCL2, antioxidant enzymes, pluripotency markers) in blastocysts, was reported previously (Barros and Paula-Lopes, 2018; Held-Hoelker et al., 2025; Kawano et al., 2025; Lang et al., 2024). Several studies revealed that blastocysts derived from heat-stressed bovine oocytes exhibit increased expression of ER stress markers (e.g., GRP78/BiP, XBP1, and ATF6 pathway genes), as well as genes associated with apoptosis and mitochondrial dysfunction (Feng et al., 2024; Held-Hoelker et al., 2025; Lang et al., 2024). Together, heat exposure during maturation disrupts the cytoskeleton, mitochondrial distribution, and calcium homeostasis and promotes apoptosis in the developing embryo (Barros and Paula-Lopes, 2018; Lang et al., 2024; Naranjo-Gómez et al., 2021).

## Molecular consequences of heat stress on early embryonic development

The severity of impediments to embryonic development at the helm of HS is largely stage-specific and primarily occurs during the pre-attachment stage, affecting cleavage and blastocyst formation, although its impact dwindles as the embryo continues in development (Ealy et al., 1993). The greatest detrimental effects to the early developing embryo occur during the pre-genome activation period (1–8 cell stage), whilst the embryo relies heavily on maternally inherited RNAs and proteins, lacking a robust stress response. For instance, early embryonic sensitivity to HS has been shown through the exposure of two-cell embryos and their subsequent rapid transcriptional increase of HSP70 Member 1A (HSPA1A), Heat Shock Protein beta-1 (HSPB1), and the synthesis of HSP70 (Khan et al., 2020; Rivera and Hansen, 2001). Additionally, embryos exposed to elevated temperatures (e.g., 40–41 °C) during early cleavage frequently exhibit reduced progression through successive mitotic divisions and significantly lowered rates of blastocyst formation, indicating compromised developmental competence and viability relative to embryos maintained at physiological temperatures (~38.5 °C) (Rivera and Hansen, 2001). Short- to moderate HS exposure during the zygote or two-cell stage of development has also been shown to disrupt cytoskeletal integrity, increase ROS, and alter mitochondrial function, all of which hinder the progression of early cleavage stage embryos through blastulation (Abdelnour et al., 2020; Al-Katanani et al., 2002; Roth and Hansen, 2004, 2005). Nevertheless, even embryos exposed to HS amid the early cleavage window are less likely to form morphologically ‘normal’ blastocysts, often exhibiting epigenetic and apoptotic changes that reflect cellular stress and reduced developmental potential, as shown by our group to be partially mitigated via practical abatement strategies such as extracellular vesicles (Menjivar et al., 2023a, 2026) and the use of antioxidants (Ramírez et al., 2026) cultured in vitro.

HS alters cell-fate decisions and subsequent implantation potential during early embryonic development via perturbations in lineage allocation, cellular survival pathways, and blastocyst quality, ultimately reducing the embryo's competence for attachment and pregnancy establishment. Exposure of early embryos to elevated temperatures induces cellular damage and oxidative stress that are known to decrease blastocyst total cell number, with particularly pronounced reductions in trophectoderm (TE) cells (Sakatani et al., 2004, 2007, 2008, 2012), the lineage responsible for placental formation and implantation (Sakatani, 2017). Additionally, HS-induced apoptosis via caspase-dependent pathway activation duly alters the balance between surviving blastomeres, undermining lineage allocation in a developmentally regulated manner (Paula-Lopes and Hansen, 2002). In addition, HS exposure during early embryogenesis has also been shown to induce abnormal chromatin remodeling and epigenetic modifications (e.g., altered H3K9me3 accumulation and heterochromatin organization), which have the capacity to reprogram gene expression patterns that govern differentiation of the inner cell mass (ICM) and TE lineages, thereby affecting downstream developmental trajectories and embryonic competence for implantation (Camargo et al., 2019). In early cleavage heat shock, it has also been shown that past a reduced blastocyst yield, further alterations in mitochondrial function and DNA integrity persist, suggesting selective survival of metabolically altered embryos with potential impacts on their post-transfer viability (Carvalheira et al., 2024). Collectively, such efforts to reshape cell-fate decisions, affecting embryonic lineage composition and the molecular programming of the early embryo, are determinants that, in part, compromise pregnancy establishment, increasing early embryonic loss in cattle.

A prime determinant governing the earliest stages of embryonic development is the supraphysiological milieu within the oviduct and uterine environments. Particularly, HS disrupts the composition of oviductal and uterine secretions (histotroph), reducing concentrations of key embryotrophic factors such as insulin-like growth factor-1 (IGF-1), colony-stimulating factor-2 (CSF2), amino acids, and antioxidants that aid in sustaining embryo metabolism and survival (Sakatani, 2017). Additionally, HS increases ROS production in reproductive tract epithelial cells, leading to oxidative damage, impaired mitochondrial function, and decreased antioxidant capacity in luminal fluids, thereby indirectly exposing developing embryos to cellular stress (Sakatani, 2017; Wolfenson and Roth, 2019). Additionally, thermoregulatory blood flow redistribution reduces uterine and ovarian perfusion, limiting tissue oxygenation and impairing oviductal transport and endometrial preparation for implantation (Wolfenson and Roth, 2019). However, mechanisms to study such undertakings in vitro are largely stunted due to the inaccessibility of physiological organs and inherent drawbacks, including critical restrictions on the formation of vivo tissue-like structures and long-term physiological function when cultured in 2D. Thus, our group specifically has made strides in using organoid models to better understand the molecular undertakings of the bovine oviduct to HS (Menjivar et al., 2023b), mapping the architecture of in vitro organoid-derived EVs with those collected in vivo and from in vitro cells in 2D and elucidating their subsequent impact on embryos developed under conditions of HS (Menjivar et al., 2026).

## Conclusions

HS is an escalating environmental challenge with profound consequences for female reproductive performance, particularly through its detrimental effects on ovarian and follicular function, oocyte maturation, and early embryonic development. This review summarizes the current state of the art on the physiological and molecular impacts of HS on ovarian function, oocyte competence, and early embryo development. Moreover, it highlights key mechanisms underlying HS-induced damage at the organ, cellular, and molecular levels, enabling the identification of potential targets for intervention. In addition to various pharmacological strategies, emerging molecular approaches, including EV-mediated signaling, offer promising avenues to enhance cellular resilience in oocytes and embryos.

## Data Availability

No research data is used.
